# Exploring Factors Influencing COVID-19 Vaccine Hesitancy and Refusal: A Study in Italy during the Vaccine Rollout

**DOI:** 10.3390/ijerph21030331

**Published:** 2024-03-12

**Authors:** Arianna Barazzetti, Stefano Milesi, Attà Negri

**Affiliations:** Department of Human and Social Sciences, University of Bergamo, 24129 Bergamo, Italy; stefano.milesi@unibg.it (S.M.); atta.negri@unibg.it (A.N.)

**Keywords:** COVID-19 vaccine, conspiracy thinking, core beliefs, trust

## Abstract

The availability of an effective vaccine against COVID-19 virus marked a crucial moment in the fight against its pandemic spread. Although distribution of the vaccine began in December 2020, high acceptance rates and repeated administrations are needed to achieve widespread immunization, but hesitation toward the vaccine persists to this day. To identify psychological variables and other factors associated with vaccine hesitancy, we conducted a study from August 2021 to October 2022. An Internet-based survey gathered data from 137 Italian adults, exploring attitudes, sociodemographic characteristics, psychological variables, and immunization behavior. The results analysis showed that gender (69.2% of vaccine-adverse people were males), education (years of education was negatively correlated with vaccine hesitancy), and religion (not declaring oneself religious or atheist was more likely to be associated with hesitancy toward the vaccine) were the variables influencing attitudes toward the vaccine. Other psychological variables differentiated people with opposite attitudes toward the vaccine: high scores on the Conspiracy Mentality Questionnaire (CMQ) and Core Belief Inventory (CBI) were positively correlated with vaccine hesitancy, indicating that individuals with more pronounced core belief violation, due to the pandemic, tend to express higher levels of vaccine hesitancy. Finally, a linear regression analysis confirmed the role of participants’ conspiracy mentality as a valid predictor for vaccine hesitancy.

## 1. Introduction

COVID-19 vaccines are being developed by many countries, and the start of the vaccination was a beacon of hope for normal life to return after the SARS-CoV-2 pandemic spread. In November 2020, Moderna and Pfizer-BioNTech reported that their much-anticipated vaccines demonstrated high effectiveness in protecting people from the COVID-19 disease [1,2]. Vaccine distribution began on 8 December 2020. 

The success of COVID-19 vaccination programs is heavily dependent on attaining broad acceptance and complete coverage. A comprehensive worldwide survey covering 19 countries showed that a significant 71.5% of respondents were willing to receive the COVID-19 vaccine if its safety and effectiveness were scientifically confirmed [3]. However, there remains a portion of the population who either refuse or hesitate to receive vaccinations, despite widespread vaccination efforts. According to the Strategic Advisory Group of Experts on Immunization (SAGE), vaccine hesitancy encompasses the phenomenon of individuals or groups showing reluctance or outright refusal to undergo vaccination, even in the face of widespread availability of vaccination services [3]. The issue of vaccination hesitancy is influenced by a multifactorial interplay, encompassing concerns about safety, effectiveness, religious and philosophical beliefs, and misinformation. In 2019, the World Health Organization (WHO) recognized vaccine hesitancy and refusal as critical hazards to international public health [4]. These obstacles not only obstruct the attainment of herd immunity but also compromise worldwide attempts to mitigate the spread of the virus. Understanding and tackling the root causes of vaccine hesitancy is crucial for the success of global immunization campaigns in the ongoing battle against the pandemic.

In general, vaccine hesitancy and refusal occur for a range of reasons: religion, culture, socioeconomic historical influences, previous experience with vaccination, communication and media, psychological aspects, and perception of vaccine safety and risks [5,6]. Previous research has shown that vaccine hesitancy is a common phenomenon globally, with variability in the cited reasons behind vaccination refusal [7]. Various studies have explored the factors impacting COVID-19 vaccine hesitancy. Handebo et al. (2021) identified several key factors that affect schoolteachers’ intention to receive the vaccine, including religious affiliation, education level, perceived susceptibility, benefits, barriers, and cues to action [8]. Larson et al. (2014) classified 147 factors that affect vaccine decisions into three categories: cognition and decision making, social norms, and communication and engagement [6]. Wang et al. (2019) found that there is a high demand for vaccination in China, but safety concerns hinder universal uptake. Their study identified several factors that influence vaccine acceptance, including gender, marital status, perceived infection risk, vaccination history, belief in effectiveness, and doctor recommendations. In contrast, the obstacles included cases in the local area, ease of vaccination, and cost considerations [9]. In Indonesia, Yanto et al. identified variables such as COVID-19 testing frequency, smoking status, and agreeableness as significant factors that influence vaccine acceptance or refusal. The decision to vaccinate in Indonesia is influenced by trust in the government and scientists [10]. Other studies have indicated that COVID-19 vaccine hesitancy has been associated with mistrust in science, governments, and health systems [11,12,13,14,15]; low perception of risk or the belief that the risks related to the COVID-19 pandemic had been exaggerated by the media; poor perception of government safety measures [12,13,16,17,18,19]; and female gender [16,20,21,22,23,24,25]. Several studies have revealed contrasting results in relation to educational level. On one hand, some studies found that vaccine refusal was more diffused among those with a university education degree [14,18], while other publications associate it with lower education [12,16,23,26,27]. 

Based on the literature presented, addressing vaccine hesitancy requires a multifaceted approach that includes public health education, targeted communication tactics, and working with communities to clarify myths and misunderstandings about vaccines. It is crucial to understand and tackle the root causes of vaccine hesitancy to ensure the success of global immunization campaigns in the ongoing fight against the pandemic. The objective of this study is to investigate the factors linked to vaccine resistance and hesitancy during the vaccination campaign in Italy. During the research period, which spanned from August 2021 to October 2022, almost 50 million people in Italy received the full COVID-19 vaccination, which represents 84% of the population [28]. Italian authorities proposed a fourth vaccine dose in the spring of 2022 to boost immunity considering the changing pandemic situation. However, there were concerns about vaccine hesitancy and achieving widespread coverage. Virus evolution and the emergence of new variants add complexity to immunization strategies. Projections suggest a 90% vaccination threshold for herd immunity [29]. Addressing hesitancy is crucial. Italy’s comprehensive strategy includes measures such as accessible vaccination options, awareness campaigns, and strict measures for non-vaccinated workers. To ensure sustained protection against the dynamic nature of the virus, ongoing public health initiatives, clear communication, and adaptable vaccination campaigns are essential. However, challenges related to vaccine refusal and hesitancy persist.

While several studies have sought to identify factors associated with COVID-19 vaccine hesitancy, they were conducted prior to the campaign of COVID-19 vaccines [8,13,21,30,31,32]. Instead, the source population for the present study is the Italian population during the vaccination campaign. 

We aimed to identify the psychological determinants, personality traits, and other factors associated with vaccine hesitancy and refusal during the vaccine rollout in Italy, from August 2021 to October 2022. It is important to identify and understand whether psychological and behavioral factors have the same importance when the outcome is real rather than hypothetical. Doing so helps to account for the psychological aspects of public health and to target campaigns and interventions for maximum efficiency. Data for the current study were collected during the vaccine campaign in Italy. The participants were asked about their acceptance of COVID vaccine and the reason for this choice. Additionally, we focused on psychological variables, personality traits, peer influence, the influence of the COVID-19 pandemic on their life and beliefs, and belief in conspiracies. By studying the multiple levels of influence on our behaviors, we can reach a more complete understanding of vaccine hesitancy. 

## 2. Materials and Methods

### 2.1. Procedures and Participants

An Internet-based survey was conducted in Italy between August and October 2021.

The survey was conducted using the Google Forms platform, and the survey link was distributed to initiate a recruitment process utilizing a snowball sampling strategy. The process commenced by targeting specific public social media pages on platforms such as social networks (e.g., from local pages frequented by residents of small towns around Milan). Notably, the survey was never directly shared by the researchers on social media platforms explicitly associated with anti-vaccine sentiments or activities. Participants were given the option to participate and were presented with a comprehensive consent form outlining the study’s objectives and emphasizing the protection of privacy. Prior to completing the questionnaires, participants were required to explicitly agree to uphold ethical standards. The average administration time was 20 min. To be included in the study, participants needed to be over 18 years of age. 

The study population included a total of 137 individuals who completed the whole questionnaire. All the participants were Italian, and the majority of them resided in the northern part of Italy (94.89%), while others resided in the central (1.46%) or southern (3.65%) parts of the nation. The sample was gender-balanced: 56.9% of the participants were females (their mean age was 41.5, SD = 11.9) and 43.1% of them were males (their mean age was 41.4, SD = 12.2). 

Even though the questionnaire had a limited circulation, probably due to the large number of its items, the data collection period was deliberately not extended beyond October 2022, so that the responses would remain representative of the specific phase of the pandemic, in which the vaccination campaign was at its peak. The number of responses remained sufficient to reach an acceptable statistical power, as this sample size allowed us to reliably investigate (*p* < 0.05, power level of 80%), correlations as low as 0.23 (Pearson’s r). The calculation is based on the formula N = [(Zα + Zβ)/C]2 + 3, for two-tailed tests.

The study followed the ethical guidelines outlined in the Helsinki Declaration, Italian law’s requirements for privacy and informed consent (Law Decree DL-101/2018), EU regulation 2016/699, and APA guidelines. All procedures were reviewed and approved by the authors’ university ethics committee. 

### 2.2. Measures 

The online survey included elements of the protocol proposed by Byrne et al. [33] for previous influenza research. The protocol consisted of questions related to participants’ health, beliefs/attitudes, and behavioral intentions toward the vaccine. 

The study examined whether participants had ever received a COVID-19 diagnosis or had experience working with infected patients. We also investigated coping strategies during the pandemic, including psychological and pharmacological therapy.

Additionally, questionnaires were utilized to evaluate various constructs such as the participant’s conspiracy mentality, personality traits, overall wellbeing, and the violation of their core beliefs during the pandemic. 

The subsequent paragraphs describe the fundamental variables included in our study and the instruments used to assess them.

#### 2.2.1. Attitude toward the Vaccine

Participants’ attitude toward the vaccine was evaluated by a specific item in the questionnaire, targeting their opinions about the serum, from most favorable to the most adverse, on a 7-point scale (Table 1).

#### 2.2.2. Other Psychological Variables and Sociodemographic Data

The sociodemographic data collected consisted of gender, age, place of residence, occupation status, religion, education level, and personal experience with COVID-19. Immediately after collecting the sociodemographic data, the participants were administered the following in sequence:

- Conspiracy Mentality Questionnaire (CMQ) [34], to measure conspiracy mentality. In our research, we used the condensed format of the CMQ, specifically the 5-item version. Participants were asked to indicate their agreement or disagreement with five statements, for example: “Many events are the result of covert groups’ schemes”. Bruder and colleagues [34] developed and validated the CMQ as a means of measuring the psychological phenomenon of conspiracy mentality. The CMQ aims to evaluate individuals’ inclination to support conspiracy theories and beliefs, offering useful insights into their cognitive and attitudinal patterns toward conspiratorial thinking.
- Big Five Inventory-Short Version (BFI-10) [35], for personality traits. The BFI-10 is a widely used personality assessment tool based on the Big Five personality traits, also known as the Five-Factor Model (FFM), which categorizes personality traits into five major dimensions. These dimensions include openness to experience, conscientiousness, extraversion, neuroticism and agreeableness.
- Wellbeing was assessed through the Personal Health Questionnaire (PHQ-4) [36]. The PHQ-4 is a widely employed self-report tool for scrutinizing anxiety and depression indicators. It includes four questions and is a condensed version of the original PHQ-9, which assesses depression more exhaustively. The PHQ-4 includes two queries related to anxiety symptoms and two related to depressive symptoms. Participants are requested to evaluate the frequency of their experiences in the past fortnight, on a scale ranging from 0 (not at all) to 3 (nearly every day). Four questions assess the following: “during the past two weeks, have you frequently experienced feeling down, depressed, or hopeless?” “During the past two weeks, have you frequently experienced a lack of interest or pleasure in carrying out activities?”
- For anxiety symptoms: “Over the past two weeks, have you felt nervous, anxious or on edge?” “How frequently have you been unable to stop or control worrying?”
- Core Belief Inventory (CBI) measured the violation of core beliefs after “the coronavirus pandemic”, which was used as the “targeted event” in the administration [37]. This survey consists of nine questions intended to assess the extent to which a person’s fundamental beliefs about the nature of the universe (in terms of fairness and controllability), the predictability of the future, the existence of a purpose in life, self-esteem, identity, and spirituality or religion have been undermined. Participants assessed the effect of a “targeted event” on a 6-point rating system, varying from “no impact” (0) to “a significant impact” (5). To ensure consistency in participants’ reflections, the generic term “targeted event” was modified to “the coronavirus pandemic“ throughout this study. High scores on the rating scale imply a considerable breach of fundamental beliefs.

### 2.3. Data Analysis

All data obtained from the survey questionnaire underwent coding and analysis using the 2021 version of the Jamovi software, an open-source statistical tool [38]. For the categorical variables (gender, religion, profession, previous COVID infections, psychological/pharmacological therapy), differences between participants with a favorable opinion about the vaccine (Likert score < 3 on item 16) and those with a negative opinion (Likert score > 3) were monitored through chi-squared tests. The six participants who did not indicate a clear attitude toward the vaccine were excluded from this computation. The rationale behind dichotomizing vaccine hesitancy in the chi-squared calculation was to mitigate the test’s sensitivity and thereby minimize the likelihood of detecting differences among groups with limited conceptual relevance. Subsequently, the chi-squared results underwent additional elucidation through a post hoc analysis of residuals, selectively applied to variables that exhibited significant disparities between the favorable and adverse groups. Later, we also performed a correlation analysis to evaluate the associations between vaccine hesitancy and the dimensional variables in our dataset, including both psychological (results from CMQ, CBI, BFI-10) and sociodemographic variables (age, years of education). Finally, a multivariable linear regression analysis was performed to evaluate a predictive model for vaccine hesitancy based on the dimensional variables included in the questionnaire and studied in the correlation analyses. Categorical variables were excluded from this model to preserve the dimensionality requirements of the test. Thus, the final multivariable regression model comprised both psychological and sociodemographic predictors of dimensional type.

For the correlation and regression analyses, vaccine hesitancy was considered not in dichotomous terms but in terms of all 7 dimensions of the Likert scale on which it was expressed by participants, in order to include entirely in the calculations the variance observed from item 16 (Table 1).

## 3. Results

The descriptive analyses revealed that participants in our sample had 16.3 years of education (SD = 3.43) on average. Most of them were favorable toward the vaccine (90.51%). Sixteen percent of the subjects had already been tested for COVID-19 virus. Furthermore, most participants declared themselves religious (63%), followed by atheist/agnostic (34%), and the rest (6%) declared themselves neither religious nor atheist/agnostic. Forty-seven percent of the participants in our sample were married. Finally, 82% of them had never worked with COVID-19 patients. 

The chi-squared analysis (Table 2) revealed that marriage, education, profession, previous COVID-19 diagnoses, reception of psychological support during the pandemic, and use of pharmaceutical support during the pandemic did not differ between the favorable group and the one adverse to vaccines. However, gender (*p* = 0.049) and religion (*p* = 0.007) were unequally distributed between the groups. 

Female subjects declared a positive attitude regarding the vaccine more frequently than males (z _adjusted_ = 1.97, *p* = 0.049), as indicated by the post hoc analysis of residuals. Similarly, those who were undecided about their religious affiliation, or did not want to declare it, were typically more favorable toward the vaccine than those who recognized themselves in a definite position, whether it was a religious or an atheist position (z _adjusted_ = 3.00, *p* = 0.0027). 

The correlation analysis (Table 3) revealed that, among the psychological dimensional variables included in our study, the *Conspiracy Mentality Questionnaire* (CMQ) score was positively correlated with participants’ vaccine hesitancy (r = 0.039, *p* < 0.001). Similarly, a positive correlation was found between vaccine hesitancy and the impact of the pandemic on participants’ *core beliefs* (r = 0.25, *p* = 0.004). 

Neither the participants’ *personality traits* nor *their level of psychological health* (PHQ-4) had a significant association with their opinion about the vaccine. 

Among the two sociodemographic indicators included in the analysis, only *participants’ education* showed an association with vaccine hesitancy (r = −0.19, *p* = 0.03); no association was found regarding *participants’ age*. 

Finally, the multivariable linear regression analysis generated a significant model (R = 0.40, *p* = 0.03) accounting for 16.3% of the observed variance in vaccine hesitancy (Table 4). 

Despite the global significance of the model, only one of its psychological components emerged as a statistically significant predictor: participants’ conspiracy mentality (*p* = 0.013), suggesting the possibility of an interaction effect between the other psychological variables in predicting vaccine hesitancy. Similarly, the sociodemographic characteristics *years of instruction* and *age* were not significant predictors of vaccine hesitancy in this multivariable model.

## 4. Discussion

Our study has identified some key factors associated with acceptance or refusal of the COVID-19 vaccine. These factors comprise both sociodemographic features and personal beliefs. 

Gender was the first sociodemographic variable that differed between those with a favorable opinion about the vaccine and those who were averse to it, as male subjects in our sample were more frequently averse to the vaccine. This result, though, needs further investigation, since contrary evidence from various studies has proposed a potential link between vaccine hesitancy and the female gender [14,16,17,20,21].

Religion was also an important sociodemographic factor that distinguished the two groups, as people confused about their religiosity, or who did not want to declare it, were frequently adverse toward the vaccine. This result suggests that those who adopt a thoughtful and indecisive attitude toward religion, without adopting the dogmas of a religious affiliation or the fixed positions of atheism, are also more wary of vaccines. 

Additionally, our research reveals that factors such as having worked with COVID-19 patients, undergone psychological therapy, taken psychiatric drugs, or been diagnosed with COVID-19 do not show significant associations with vaccine stance. However, it is imperative to note that while the data on marital status, occupation, receipt of psychological support during the pandemic, and use of pharmaceutical support during the pandemic are consistent with previous research [16,17,21], the association between vaccine stance and the contagion of COVID-19 warrants further investigation, as it challenges prior findings suggesting that individuals who experienced severe symptoms of COVID-19 exhibited more hesitancy toward vaccination than those who did not encounter the disease at all [12].

Regarding the role of instruction in shaping vaccine adversity, our results are mixed. On the one hand, education does not reach the level of significance as a predictor of vaccine hesitancy; on the other hand, a significant negative correlation is observed between the two variables (r = −0.19, *p* = 0.03). The literature has already discussed the possibility that lower educational attainment might be associated with inadequate health literacy, impacting the ability to acquire, process, and comprehend crucial health information necessary for making informed decisions [39]. This deficiency could potentially lead to confusion and uncertainty, diminishing the inclination to undergo vaccination, if proper informative campaigns are not organized for the less educated part of the population. This hypothesis, though, requires further research. 

Based on the findings of other authors [32,40] and on our own research, a pre-existing diagnosis of anxiety or depression before the pandemic did not affect willingness to take the vaccine. The mental health indicators considered in our study do not show any association with vaccine hesitancy: the attitude toward the serum appeared independent from depressive or anxious symptomatology. This result, along with the already mentioned observation that the experience of psychological or pharmaceutical therapy does not influence vaccine hesitancy, suggests that psychopathology and vaccine hesitancy are independent constructs. Thus, during vaccination campaigns, it may not be necessary to concentrate additional efforts to reach people with psychopathology. 

The results from the personality traits assessment (TIPI) suggest no association between personality and vaccine hesitancy, indicating that personality may not significantly contribute to shaping vaccine attitudes in the studied population. This result is particularly important since the relation between personality and vaccine hesitancy is still uncertain. For example, a study by Weikl and colleagues [41] highlights a significant correlation between vaccine acceptance and only the trait of openness to experience, and another by Viskupič [42] and collaborators shows a correlation between vaccine acceptance and only the trait of agreeableness. Both studies, though, show a small correlation between these constructs, and the data observed in our sample is fully consistent with the idea that there is no correlation, or a very small one, between vaccine hesitancy and personality traits. 

The *Conspiracy Mentality Questionnaire* total score exhibits a substantial positive correlation (r = 0.39, *p* < 0.001) and emerged as a significant predictor in the multivariate analysis, indicating a noteworthy relationship between a tendency toward conspiracy thinking and higher vaccine adversity. These data suggest that individuals with higher scores on the CMQ, reflecting a greater tendency to endorse conspiracy theories, exhibit a more adverse attitude toward COVID-19 vaccination. The result emphasizes the role of distrust in official information and narratives in influencing vaccine hesitancy. This finding is consistent with previous studies showing an association between COVID-19 vaccine hesitancy and belief in conspiracy theories [12,16,23] and lack of trust in institutions or the government and scientists [11,12]. In addition, previous research has shown that conspiracy theories can damage trust in authorities and institutions [16] and act as a barrier to health-protective behavior, including unwillingness to vaccinate [12,43,44].

Finally, the analysis of the relationship between CBI score and vaccine hesitancy shows mixed results. Although the CBI score demonstrated a significant correlation with vaccine hesitancy (r = 0.25, *p* < 0.01), in our regression model it did not reach the level of significance necessary to be considered a valid predictor. This result needs further clarification; one possibility is that the impact of the pandemic on people’s *core beliefs* contributes to vaccine hesitancy only in the interaction with other variables [45,46]. If, on the other hand, a direct relationship is confirmed, vaccine hesitancy could be considered a form of defensiveness in response to the stressful event of the pandemic; the most stressed individuals would be the ones more prone to hostility toward the vaccine. This defensive stance could serve to uphold a sense of control or security amid the tumultuous circumstances and uncertainties associated with the ongoing health crisis.

Overall, our findings underscore the multifaceted nature of factors influencing vaccine adversity, encompassing educational background, conspiracy thinking, personality traits, and core beliefs. These insights can be valuable for tailoring interventions and communication strategies to address diverse concerns related to vaccine acceptance.

### Limitations and Future Research Directions

The limited duration of the data collection process is both a strength and a potential weakness of the gathered data, making them clearer to analyze but less generalizable to different phases of the vaccinal campaign. 

In particular, the study was conducted between August 2021 and October 2022 in Italy, thus capturing only a specific period of the vaccination campaign, which roughly coincided with its most intense phase. The limited duration of the data collection period also impacted negatively on the size of the sample employed in the study, especially exposing the results to an increased risk of Type-1 and Type-2 errors. 

Our findings regarding the correlations between both CBI and CMQ scores with vaccine hesitancy could be considered reliable since they fall within the range defined acceptable by the statistical power test relative to our sample (as discussed in Section 2.1). Regarding the chi-squared test on the distribution of adverse and favorable participants in the groups defined by profession, working with COVID-19 patients, and religiosity, the results should be considered with caution because of the very small number of participants who expressed uncertainty about their religiosity in our sample. This weakness in sampling may explain the difference between our results and those of Milligan and colleagues [47]; indeed, the latter found a positive correlation between spirituality and vaccine acceptance, specifically indicating lower vaccine acceptance among highly religious individuals. Due to the predominant representation of respondents from northern Italy in our study cohort, our findings offer valuable insights specific to this region. However, it is essential to acknowledge the inherent limitations of this narrow focus and recognize that the conclusions drawn may not be directly applicable to other regions within Italy, the entire Italian population, or other countries. The differential impact of the COVID-19 pandemic across northern and southern Italy underscores variations in population density, healthcare infrastructure, socioeconomic factors, and governmental responses. These regional disparities may similarly manifest in other countries. To mitigate this limitation, future research endeavors should prioritize expanding the study’s scope to include a more diverse sample, thereby enhancing the generalizability of the findings across different geographical areas. Furthermore, the use of an Internet-based survey introduces potential selection bias, as respondents might not be representative of the broader population. Those without Internet access or with different attitudes toward vaccination might be underrepresented. Additionally, the study does not extensively delve into external factors, such as media influence, political climate, or global events, which can significantly impact public attitudes toward vaccination. These factors are dynamic and may change rapidly, affecting the study’s relevance over time. Exploring potential future avenues of research involves embarking on longitudinal studies to meticulously track the different shifts in vaccine attitudes over time. This complex approach can provide a comprehensive and detailed exploration of how attitudes evolve and change in the dynamic landscape of an ongoing vaccination campaign.

To enrich the findings, a complementary strategy could involve augmenting quantitative data with qualitative insights gathered from interviews. This methodological fusion offers a comprehensive understanding of the various motivations influencing vaccine attitudes, uncovering subtleties that quantitative measures alone may overlook.

## 5. Conclusions

Our study sheds light on the critical factors influencing the acceptance or refusal of COVID-19 vaccination during the vaccine rollout in Italy from August 2021 to October 2022. The findings underscore the intricate interplay of sociodemographic features, personal beliefs, and attitudes toward vaccination in shaping individuals’ decisions. 

Participants with a higher conspiracy mentality and those experiencing a significant impact on personal core beliefs during the pandemic exhibited a more adverse attitude toward vaccination. Additionally, the inverse relationship between higher education levels and vaccine acceptance suggests the need for targeted interventions tailored to different educational backgrounds. While our research contributes to the evolving understanding of vaccine hesitancy, it is crucial to acknowledge the dynamic nature of public sentiment. Ongoing research and adaptive public health strategies are essential to effectively navigate the evolving landscape of COVID-19 vaccine acceptance and refusal. 

It is essential to assess public sentiment regularly, scrutinize vaccine coverage rates, and adapt strategies based on real-time data in order to remain responsive to evolving situations. By combining regulations with specific educational initiatives and community involvement, the governing bodies can establish a more holistic and efficient strategy to tackle vaccine reluctance and hesitancy, ultimately contributing toward the objective of achieving widespread immunization coverage in this and other healthcare emergencies.

## Figures and Tables

**Table 1:** Item 16: “What is your opinion about the COVID-19 vaccine?”.

Participant’s Opinion	Likert Score	Participant’s Answer
Very positive	0	COVID-19 vaccines are the main weapon against the pandemic
Positive	1	COVID-19 vaccines are a useful and safe health aid
Vigilant	2	COVID-19 vaccines may be useful, but there is not a precise clinical validation
Neutral	3	I have no opinion about this
Critical	4	COVID-19 vaccines don’t have a real scientific basis
Negative	5	COVID-19 vaccines are more harmful than helpful
Very negative	6	COVID-19 vaccines are the result of a dangerous commercial and political operation

**Table 2:** Differences in the categorical variables between participants with favorable and adverse opinions about the vaccine. Values are rounded to two significant digits.

Sociodemographic Data	Favorable *n* = 118	Adverse *n* = 13	Χ^2^	*p*-Value
Gender	n (%)	n (%)	3.88	0.049 *
Female	70 (95%)	4 (5%)		
Male	48 (84%)	9 (16%)		
Marriage status				
Married (or cohabitant)	53 (87%)	8 (13%)	1.30	0.25
Others (single, divorced, widowed)	65 (93%)	5 (7%)		
Religion			9.92	0.007 *
Atheist/agnostic	42 (96%)	2 (5%)		
Religious	72 (90%)	8 (10%)		
Confused/did not indicate	4 (57%)	3 (43%)		
Educational Status			3.94	0.27
Middle school and below	4 (67%)	2 (33%)		
Diploma	39 (91%)	4 (9%)		
Degree	57 (92%)	5 (8%)		
PhD	18 (90%)	2 (13%)		
Job category			4.12	0.77
Health worker	28 (90%)	3 (10%)		
Teacher or researcher	16 (94%)	1 (6%)		
Office employee	46 (90%)	5 (10%)		
Manual profession	4 (80%)	1 (20%)		
Social field	3 (100%)	0 (0%)		
Manager	7 (100%)	0 (0%)		
Student	2 (67%)	1 (33%)		
Other	12 (86%)	2 (14%)		
Have you ever worked with COVID-19 patients?			0.22	0.64
Yes	21 (88%)	3 (13%)		
No	97 (91%)	10 (9%)		
Did you receive psychological therapy during the pandemic?			1.28	0.26
Yes	21 (84%)	4 (16%)		
No	97 (92%%)	9 (9%)		
Did you take psychiatric drugs during the pandemic?			0.00	
Yes	9 (90%)	1 (10%)		0.99
No	109 (90%)	12 (10%)		
Have you been diagnosed with COVID-19?			0.53	
Yes	18 (86%)	3 (14%)		0.47
No	100 (91%)	10 (10%)		

* *p* < 0.05.

**Table 3:** Pearson correlations for dimensional study variables in the sample population. Values are rounded to two significant digits.

Variable	Correlation with the Vaccine Adversity Score	*p*-Value
Age	−0.018	0.96
Instruction (years)	−0.19	0.030 *
CMQ (total score)	0.39	<0.001 **
TIPI		
Extraversion	0.058	0.50
Openness	0.070	0.42
Conscientiousness	0.080	0.35
Agreeableness	−0.039	0.65
Neuroticism	0.12	0.18
PHQ-4		
Anxiety	0.13	0.14
Depression	0.059	0.49
Total score	0.10	0.23
CBI (total score)	0.25	0.004 **

* *p* < 0.05, ** *p* < 0.01.

**Table 4:** Multivariable linear regression predicting vaccination hesitancy based on psychological and sociodemographic characteristics. The global test of the model is statistically significant (F = 2.05, *p* = 0.03). Values are rounded to two significant digits.

Predictor	β Estimate	t	Standard Error	*p*
Intercept	−0.075	1.20	−0.062	0.95
CBI (total score)	0.17	0.11	1.57	0.12
CMQ (total score)	0.14	0.054	2.51	0.013 *
PHQ				
PHQ-4_anxiety	0.0048	0.092	0.052	0.96
PHQ-4_depression	−0.15	0.086	−1.79	0.077
TIPI				
Extraversion	0.0077	0.036	0.21	0.83
Agreeableness	−0.049	0.060	−0.82	0.41
Conscientiousness	0.028	0.048	0.58	0.56
Neuroticism	0.054	0.046	1.18	0.24
Openness	0.039	0.046	0.85	0.40
Years of instruction	−0.033	0.034	−0.95	0.34
Age	−1.19 × 10^−4^	0.009	−0.013	0.99

* *p* < 0.05.

## Data Availability

Data are available on request.
